# Beyond the forests: peatlands as overlooked carbon stores in coastal British Columbia

**DOI:** 10.1038/s41598-026-44791-z

**Published:** 2026-03-19

**Authors:** Hanna Rae Martens, Juergen Kreyling

**Affiliations:** Institute of Botany and Landscape Ecology, University of Greifswald, Partner in the Greifswald Mire Centre, Soldmannstraße 15, D-17489 Greifswald, Germany

**Keywords:** Climate sciences, Ecology, Ecology, Environmental sciences

## Abstract

Peatlands are significant carbon stores, and yet their carbon stocks in coastal British Columbia, Canada, remain largely unquantified. We conducted a field assessment to estimate above- and belowground carbon stocks at six peatland sites across the coast of British Columbia. These values, together with data from the Canadian Peat Profile Database, were compared with regional aboveground carbon stock estimates of the upland forests. We found that coastal peatlands store approximately three to five times more carbon per area than adjacent temperate rainforests. Extrapolating these numbers to regional sums is hampered by lacking precise information about the peatland extent of the region. Assuming peatlands cover about 5% of coastal BC, they store at least 370 Mt C, which is about 20% of the upland forest C stock. These results underscore the importance of peatlands, and highlight a need for further assessment of peatlands in this region.

## Introduction

The Pacific Northwest of North America is known for its lush temperate rainforests, which contain about 3.58 Pg of carbon across coastal British Columbia (BC) and Alaska^1^. These carbon stocks are considerably higher than in other forests of North America^2,3^ and, in fact, among the highest for forests world wide^4^.

Generally, peatlands are highly efficient carbon stores, containing 600 ± 100 Pg of carbon globally, which is about 30% of the global soil carbon stored on just 3% of the global land area, or twice the amount of carbon as found in the entirety of Earth’s tree biomass^5^. However, peatlands of western North America are known to be poorly mapped^5^. Peatland carbon stores for this region have not yet been reported beyond global model outputs, which have suggested up to 2.5 m of peat thickness in the peatlands studied here^6^. Other studies from Northern BC have found peatland depth ranging from 1 to 2 m, with up to 3–4 m in basin bogs^7–10^. Generally, improved estimates of the depth and extent of Canadian peatlands are urgently needed^3^.

Using 195 of our own and 94 additional samples from the Peat Profile Database^11^, we set out to quantify peatland carbon stocks of the Pacific Northwest of British Columbia, Canada. This was compared with the known forest carbon stocks of the region.

## Methods

**Fig. 1:**
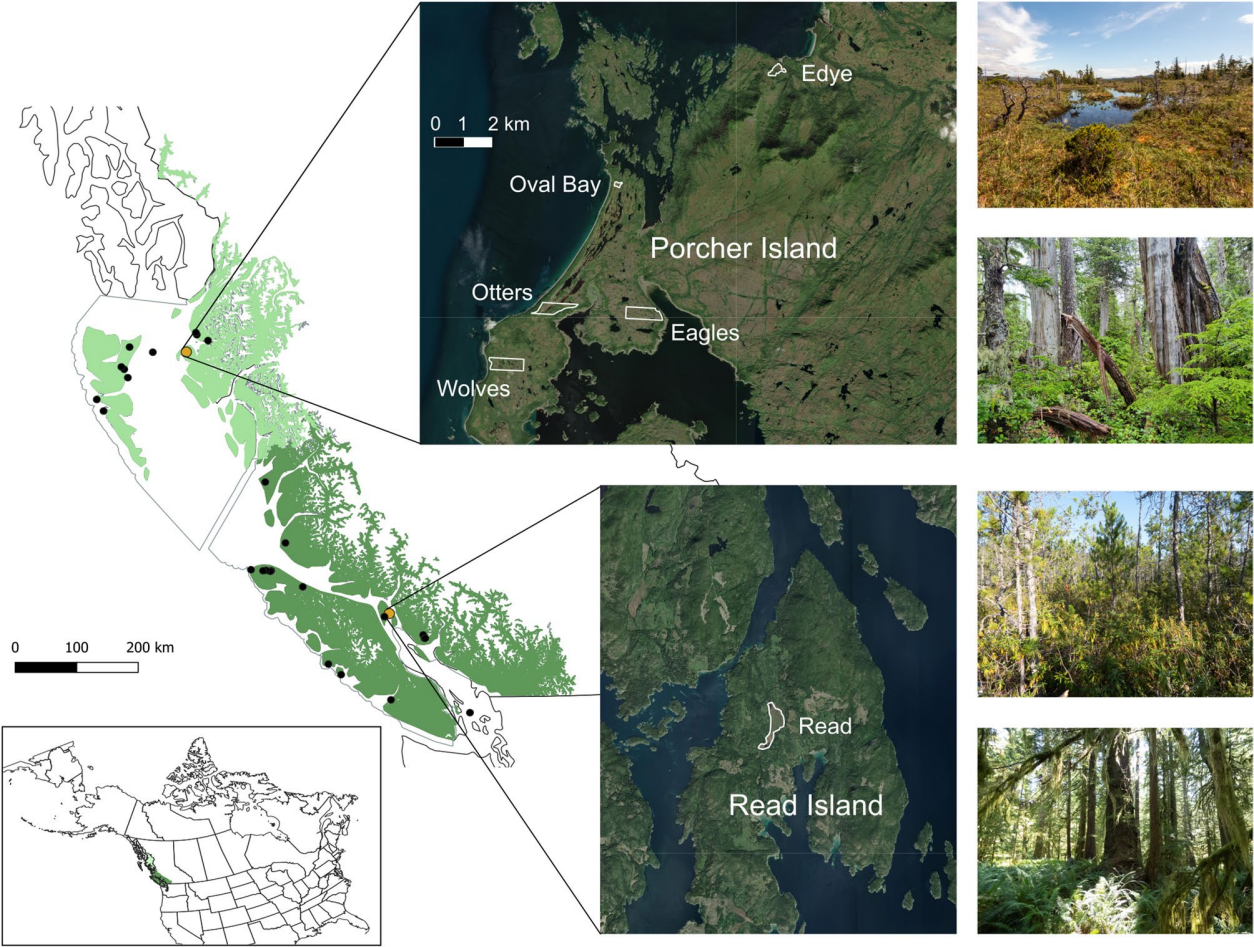
Map of the study sites across Northern (light green) and Southern (dark green) BC within the Coastal Western Hemlock Zone (all green area), including photos of typical peatlands (upper picture per region, respectively) and upland forests (lower picture per region, respectively). Our plot locations are shown in orange and the locations of points from the Peat Profile Database are in black^11^. Maps were generated using QGIS^14,15^.

We conducted the study within six peatlands located in Southern (Read Island) and Northern (Porcher Island) Coastal BC within the study region of Lamping et al.^1^ (Fig. 1). Due to accessibility and logistical considerations, sites were selected from peatlands owned and protected for long term nature conservation by the foundation Wilderness International. Additionally, Oval Bay, which is part of the Gitxaala Nii Luutiksm/Kitkatla Conservancy, was included to provide an additional site. Permission to conduct research at Oval Bay was provided by the Metlakatla First Nation. All sites are located within the hypermaritime Coastal Western Hemlock Zone (CWH), which is characterized by mean annual precipitation of 2228 mm and extensive wetlands^12^. Peat in this region has been accumulating for the last 13,500–10,500 years, since the last glaciation period^9,13^.

Potential peatland extent for each site was visually outlined in QGIS^15^ based on vegetation type using aerial imagery and classified into open peatland and forested peatland polygons. One plot per three hectares was randomly placed using QGIS^15^, stratified proportionally to open versus forested area, resulting in 5 (Oval Bay) to 15 (Eagles) plots (Table 1). Following the Field Guide for Estimating Carbon Stocks in Wetlands developed by the Quebec government^16^, each 100 m^2^ plot was divided into four subplots (25 m^2^). To estimate the soil organic carbon, the depth of the organic deposit was measured at random locations within three out of the four subplots. Decomposition status was noted based on the degree of decomposition (humic or fibric) in the middle tier (50–100 cm, or the average of the whole deposit when < 50 cm) following the Field Guide for Estimating Carbon Stocks. To determine the carbon in aboveground woody biomass, the species identity, diameter at breast height, and diameter at stump height was measured for all living trees (> 4 m) within the full plot, and for all shrubs (1.3–4 m, > 1 cm diameter) within one of the subplots, here always the south-eastern quadrant. Plant identification was conduced in the field and no plant material was collected. Therefore, the work complied with all relevant regulations and legislation.

**Table 1:** List of sites and peatland type, area, and number of plots for the open and forested areas. Peatland type is listed across all sites as bog, but Northern BC sites may potentially be poor fens^10^.

Region	Site	Peatland Type	Open Plots	Forested Plots
Northern BC	Oval Bay	Blanket bog	6	0
	Otters	Blanket bog	4	7
	Wolves	Blanket bog	6	8
	Eagles	Blanket bog	12	3
	Edye	Slope bog	4	5
Southern BC	Reed	Basin bog	5	4

Allometric equations were used to convert the diameter at breast height into the total aboveground woody biomass per plot^17^. Each species has a unique equation, but since not all species were available, the most similar species were used (*Picea mariana* for *Picea sitchensis*,* Thuja occidentalis* for *Callitropsis nootkatensis*, *Tsuga canadensis* for *Tsuga heterophylla)*. The biomass was then multiplied by the standard 50% carbon content for woody biomass^18^ to determine the total aboveground carbon stored per plot.

Carbon in the peat and regional peat C stocks were calculated based on our own samples and all cores within the CWH zone from the Peat Profile Database^11^. As our study did not include bulk density samples, bulk density values from the Peat Profile Database were used. The mean of all bulk density measurements from the CWH zone was calculated, resulting in an average bulk density of 0.14 g cm^3^ for highly decomposed, humic samples (Canadian Soil Classification System Oh horizon, 152 samples) and 0.09 g cm^3^ for low decomposition, fibric (Om horizon, 39 samples). This is slightly lower than the global average of 0.15 g cm^3^ in temperate regions^6^. These values were then applied to our own depth measurements based on the decomposition status being humic or fibric. For additional samples, the total organic depth from the Peat Profile Database was determined for the entire organic layer (SAMP_THICK) as defined by the Canadian System of Soil Classification (SAMP_OM_CSSC = ORGANIC). Here, the mean bulk density per sample was calculated. The resulting bulk density values were multiplied by the peat depth and the conversion factor of 45% carbon content for nutrient poor temperate peatlands was applied^18^.

**Fig. 2:**
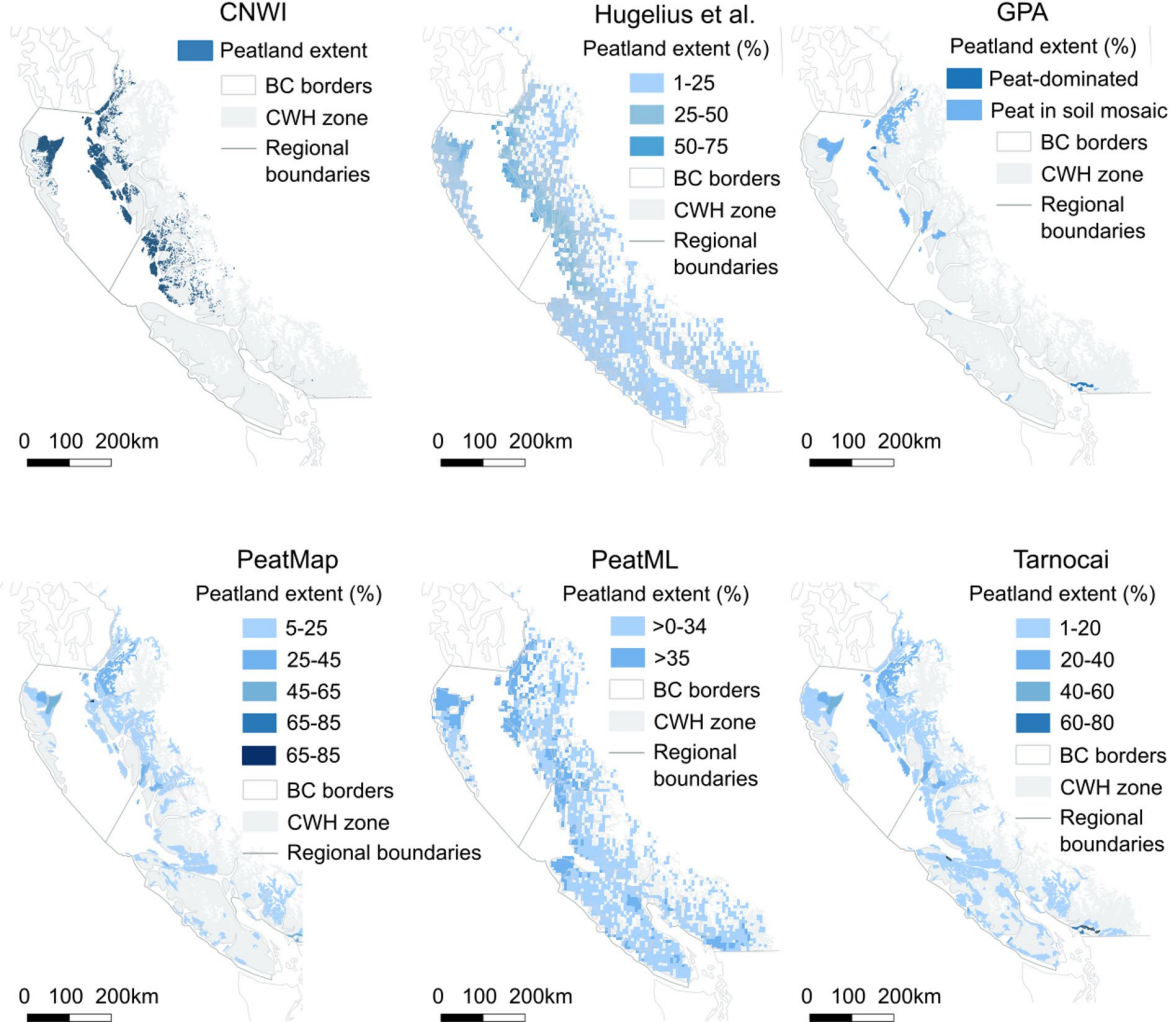
Peatland extent of the Coastal Western Hemlock zone of Northern and Southern BC (grey) varies strongly between available map products. Across all sources, increasing peatland extent is shown as darkening blue. Maps show individual peatland polygons for CNWI^19^, and peatland extent in percent for the other sources^5,20–23^. Maps were generated using QGIS^15^.

Since peatland extent estimates from this region vary between sources, multiple sources were compared. This included peatland area from (1) all bog and fen polygons from the most recent CNWI publication^19^, (2) peatland extent as a share within larger pixels by Hugelius et al.^20^, for the histosol fraction in the top 1 m of soil, (3) the peat dominated and peat in soil mosaic area from the Global Peatlands Assessment^5^, (4) PeatMap^21^, (5) PeatML^22^, and (6) the peatland area Geological Survey of Canada’s map^23^ (Fig. 2). Of these, maps (1, 3, 4) and (6) come from regional, national, and in the case of (3) and (4), global data relying on field observations. Maps (2) and (5) are machine-learning based global peat estimates. Due to insufficient spatial resolution, sources (2) and (5) were included for comparison purposes but not for further peat area calculations. Each peatland extent map was clipped to the extent of the Coastal Western Hemlock (CWH) Biogeoclimatic Ecosystem Classification Zone^24^. Using the map with the greatest peatland coverage^23^, we scaled our calculated C stock values to the extent of the peatlands reported on the Pacific Coast of BC (CWH zone). The size of the CWH zone was calculated using national census boundaries^25^ and the CWH zone extent^24^.

Finally, we compared our results to published records of forest carbon stocks^1^. Following their regionalization, we classified the North Coast and Kitimat-Stikine Regional Districts as Northern Coastal BC and the Central Coast Regional District and southwards as Southern Coastal BC to the extent they fell within the Pacific Maritime Ecozone^26^.

## Results

We studied a total of 195 cores (6 sites) and combined this with 94 cores (23 sites) from the Government of Canada’s Peat Profile Database^11^. Our study sites consisted of open and forested peatlands, with Yellow Cedar (*Callitropsis nootkatensis*), Shore Pine (*Pinus contorta var. contorta)*, and Western Hemlock (*Tsuga heterophylla*) occurring as the dominant tree species. In Northern BC, ground cover was dominated by Common Juniper (*Juniperus communis*), Caribou lichen (*Cladonia* sp.), and *Sphagnum* species, including *S. austinii*,* S. papillosum*,* and S. capillifolium*. In Southern BC, Labrador Tea (*Rhododendron groenlandicum*) and Sweet Gale (*Myrica gale*) formed the dominant shrub cover, with extensive *Sphagnum* below.

Peat thickness across all plots reached a grand mean of 105 ± 104 cm in Northern Coastal BC, yet varied strongly between a few centimetres in rocky muskegs to more than 420 cm in well developed bogs. Besides this patchiness, it is noteworthy that we found surprising peat depths of up to 150 cm even in forested (> 25% tree cover) peatlands. For Southern Coastal BC, the grand mean peat thickness was 183 ± 130 cm.

Aboveground carbon stocks in our investigated peatlands were small across all sampled plots, reaching a grand mean of 1.17 ± 1.23 kg C m^− 2^. The maximum aboveground plot level C stock was 7.88 kg C m^− 2^ (at Otters), while the open bogs had no trees or even shrubs above breast height (Table 2). This data was not available from the additional dataset.

**Table 2:** Above- (trees & shrubs) and belowground (peat) carbon (kg C m^− 2^) for each study site. Oval Bay only had one tree and therefore SD aboveground is not provided.

Site	Aboveground		Belowground	
	Mean	SD	Mean	SD
Eagles	1.21	1.18	34.72	40.19
Edye	0.92	0.50	24.29	23.46
Otters	3.54	2.79	74.78	57.43
Oval Bay	0.74	NA	72.55	56.58
Read	0.53	0.11	46.48	36.37
Wolves	1.01	0.93	82.87	45.11

**Fig. 3:**
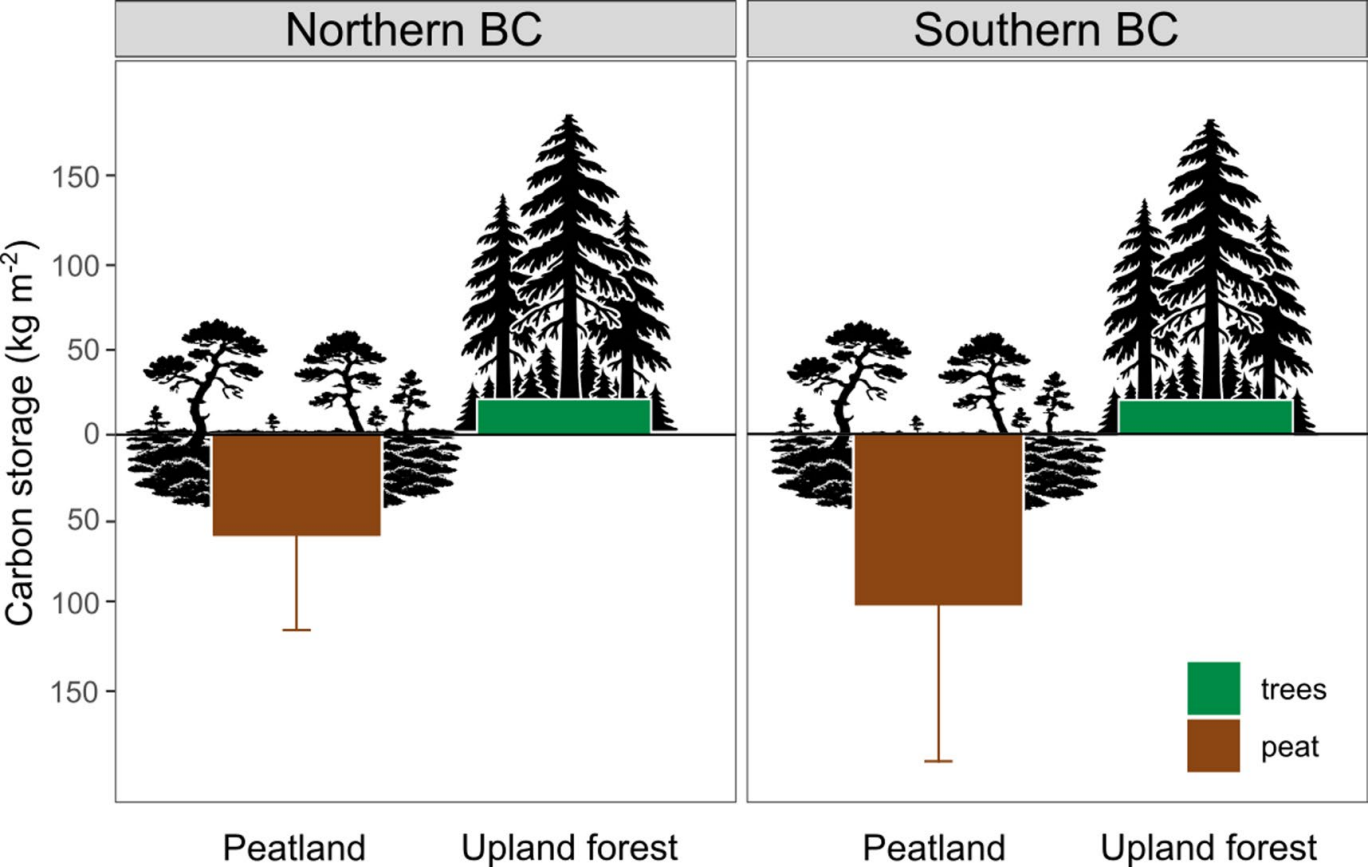
Amount of carbon stored belowground based on data from the Peat Profile Database and our own data compared to regional forest averages^1^. Mean values and SDs for Northern and Southern BC are given.

Carbon stocks within the peatlands were dominated by the peat, reaching 44 times the amount of the aboveground carbon stocks across all sites. Total carbon stocks from our data ranged from 24.29 ± 23.46 kg C m^− 2^ at Edye on a slope bog to 82.87 ± 45.11 kg C m^− 2^ in a blanket bog at Wolves (Table 2). The large SDs reflect the patchiness of the peat thickness. Peat core data from the Peat Profile Database^11^ had carbon stocks ranging from 24 to 206 kg C m^− 2^. Taken together, these values reach a mean of 58.60 ± 55.49 kg C m^− 2^ for Northern BC, and 99.02 ± 91.38 kg C m^− 2^ for Southern BC (Fig. 3).

Regional peatland extent estimates varied, from 2193 km²^19^ to 5205 km²^23^. The Global Peatlands Assessment estimates 301 km² for this region only including peatland dominant area, or 6617 km² for peat soils in mosaic^5^. These values are equivalent to between 2 and 5% of the total CWH region (107200 km²). Swamps alone cover 1707 km² of the region based on CNWI estimates, but are not included in peatland estimates since their soil type is not documented. Using a peatland coverage estimate of 5205 km²^23^ and our calculated mean regional value of 71.17 kg C m^− 2^, we estimate a regional carbon stock of 370 Mt C or 1358 Mt CO₂e. Given the estimated carbon stocks of 20 kg C m^−^², the CWH region stores approximately 2040 Mt C in the forests on mineral soil. Therefore, peatlands store 18% of this carbon while covering only 5% of the region.

## Discussion

Peatlands of the BC coastal region store on average 59 kg C m^− 2^ for Northern Coastal BC and 99 kg C m^− 2^ for Southern Coastal BC. Therefore, these coastal peatlands hold three to five times as much carbon per area as the coastal rainforests of the region, which contain a mean of about 20 kg C m^−^² in their tree biomass^1,27^. These recent numbers for the forests are higher than earlier estimates of global mapping studies for the region, reaching 10 kg C m^−^²^2^ or 7 kg C m^−^²^4^. More local estimates came to similar numbers, i.e., 10 kg m⁻² for southern Alaska^28^. While other research has demonstrated that forested peatlands within the boreal region of Canada hold substantially more carbon (22.6–66.0 kg C m^− 2^) then both the aboveground forests (2.8–5.7 kg C m^− 2^)^29^ and the surrounding forests (> 5 kg C m^− 2^)^3^, this is the first study to demonstrate the same is true even for the temperate rainforests of BC.

In comparison to other peatland rich areas of Canada, the Pacific Coast appears to contain high carbon stocks. Recent estimates have suggested that Northern hemisphere peatlands contain a mean carbon stock of 132 Gg C km^− 2^ (equivalent to 132 kg C m^− 2^)^6^ or 106 ± 66 kg C m^− 2^^20^. Other sources specifically for the Pacific Coast have estimated the carbon stocks at 60–85 kg C m^− 2^^3^ or a mean of 98.88 ± 70.82 kg C m^− 2^^23^. Our similar regional mean of 71.17 kg C m^− 2^ confirms that, while lower than other peatland regions of Canada, the peatlands of the Pacific Coast are still of national importance for carbon stocks. Interestingly, the SoilGrid250 m map shows Canada’s highest soil organic carbon stocks are within the Pacific Coast region^30^, though a similar approach but using a model specifically trained for Canada highlighted the Hudson’s Bay Lowlands as the largest soil organic carbon stock^3^.

Peat depth and carbon storage varied drastically within and across the studied peatlands, being as patchy as expected for muskegs on hilly terrain in a young landscape^9^. Yet we also found deep peat even within heavily forested areas which are often mapped as forested swamps and not as peatlands. Research by Davidson et al.^31^, has shown that swamps across North America, particularly when dominated by needle-leaved species, can be deep carbon reservoirs, and yet are generally poorly studied, especially within our study region. The most recent version of the Canadian National Wetlands Inventory contains no soil composition information for the 1707 km² of swamps in the region^19^ making it impossible to determine the contribution of this peatland type to the regional carbon stock. Indeed, Sothe et al.^3^, in an assessment of Canadian carbon stocks, notes that the Pacific Maritime region contains high amounts of soil organic carbon, likely due to the presence of peatlands and, notably, forested peatlands with high C densities in tree peat.

Surprisingly, not just forested peatlands, which are certainly harder to detect, but also areas of open peatland range substantially between published sources (Fig. 2). Especially in Southern BC, peatland extent varies dramatically between sources. Only limited peat core data from this region exists; our own samples more than doubled the number of cores that existed in the national Peat Profile Database. It is evident that the Pacific Coast, along with many other peatland rich regions worldwide, suffers from rough and outdated mapping^32^. Large variation in peatland extent of our study region between sources (Fig. 2) can likely be explained by (I) a general shortage of observational data from the region even in maps with a regional to national focus (maps 1, 6). This also results in the lack of regional precision going unrecognized in global maps that are based on observational, i.e., elsewhere published, data (maps 3, 4). Other global mapping activities have used machine learning algorithms based on climate and topographic data including remote sensing information, which appear to suffer from (II) not taking region-specific factors such as the importance of the micro- and mesotopography on precipitation sums into account in machine-learning algorithms (maps 2 and 4). Taken together, further activity is required to combine systematically sampled ground truth data in the field with regionally-focused machine-learning algorithms containing finely resolved topography and remote sensing data specifically for the Pacific Coast region.

Despite their role as hotspots of carbon storage in the landscape, peatlands lack recognition and protection^33^. Needle-leaved swamps in particular can accumulate large amounts of peat over a long time, and therefore if disturbed, it is unlikely that these carbon stores will recover^31^. Our study emphasizes the need for recognition and protection of these large carbon stores.

Future research should provide a more detailed approach combining systematic, observational ground truthing with region-specific modeling across coastal British Columbia, particularly in the areas likely to contain peat that are beyond current mapped extents. Characterization of forested peatlands would also reduce uncertainties in carbon stock estimates, as would further sampling points. Given the extreme variability in both the micro- and mesotopography of this region, more data on peat depth and density is needed to improve regional peatland modelling and quantification of carbon sequestration over time and space, e.g., by incorporating successional processes affecting ecohydrology of the peatlands.

## Conclusion

Our results reiterate the value of peatlands as carbon stores. Even in a landscape like the temperate rainforests of coastal BC, which are known for their massive stores of aboveground carbon in their trees, the peatlands of this region still hold three to five times the amount of carbon per area. Mapped peatland extent in the region has not yet converged between sources and approaches. Conservatively assuming 5% peatland cover, our data suggests that peatlands store 370 Mt C in coastal BC or about one fifth of the total carbon stock of the region. Peatlands therefore should no longer be overlooked in this region. Understanding their true extent and carbon store is essential for effective conservation and climate change mitigation strategies in British Columbia.

## Data Availability

Above- and belowground biomass data is available at [https://doi.org/10.5281/zenodo.17533263].
